# Assessing changes in quality of life using the Oral Health Impact Profile (OHIP) in patients with different classifications of malocclusion during comprehensive orthodontic treatment

**DOI:** 10.1186/s12903-015-0130-7

**Published:** 2015-11-20

**Authors:** De-Hua Zheng, Xu-Xia Wang, Yu-Ran Su, Shu-Ya Zhao, Chao Xu, Chao Kong, Jun Zhang

**Affiliations:** Department of Orthodontics, School of Dentistry, Shandong University, Jinan, Shandong Province People’s Republic of China; Department of Oral and Maxillofacial Surgery, School of Dentistry, Shandong University, Jinan, Shandong Province People’s Republic of China

**Keywords:** Oral health-related quality of life, Orthodontic treatment, Patient assessment

## Abstract

**Background:**

The objectives of this study were to investigated changes in OHRQoL among patients with different classifications of malocclusion during comprehensive orthodontic treatment.

**Methods:**

Clinical data were collected from 81 patients (aged 15 to 24) who had undergone comprehensive orthodontic treatment. Participants were classified 3 groups: Class I (*n* = 35), II (*n* = 32) and III (*n* = 14) by Angle classification. OHRQoL was assessed using the Oral Health Impact Profile (OHIP-14). All subjects were examined and interviewed at baseline (T0), after alignment and leveling (T1), after correction of molar relationship and space closure (T2), after finishing (T3). Friedman 2-way analysis of variance (ANOVA) and Wilcoxon signed rank tests were used to compare the relative changes of OHRQoL among the different time points. A Bonferroni correction with *P* < 0.005 was used to declare significance.

**Results:**

Significant reductions were observed in all seven OHIP-14 domains of three groups except for social disability (*P* > 0.005) in class I and class II, Handicap in class II and class III (*P* > 0.005). Class I patients showed significant changes for psychological disability and psychological discomfort domain at T1, functional limitation, physical pain at T2. Class III patients showed a significant benefit in all domains except physical pain and functional limitation. Class II patients showed significant changes in the physical pain, functional disability, and physical disability domains at T1.

**Conclusions:**

The impact of comprehensive orthodontic treatment on patients’ OHRQoL do not follow the same pattern among patients with different malocclusion. Class II patients benefits the most from the stage of space closure, while class I patients benefits the first stage (alignment and leveling) of treatment in psychological disability and psychological discomfort domains.

## Background

The concept of oral health-related quality of life (OHRQoL) describes the patient-perceived impact of oralfacial conditions and effect of dental interventions. It is a broad and comprehensive concept which is widely influenced by physical health, psychological state, social relationship, environment and so on. In order to evaluate it objectively, measuring instrument (OHIP-14) covering seven specific domains were originally developed and examined by Slade GD [1]. As a sensitive assessment tool, it can not only help clinicians to assess patient’s current oral state but also worked as an indicator to help researchers to supervise changes in oral health-related quality of life. For this reason, this proven approach has drawn increasing attention from research workers and clinicians in oral-related discipline. Subsequently, it was widely used by Scholars from several branches of stomatology to evaluate the impact of different therapeutic methods on oral health-related quality of life of patient. For example, Pei liu et al. [2], a prospective longitudinal study consisting of 279 patients reported that root canal therapy improve oral health-related quality of life significantly. Likewise, Viola AP et al. [3], found that conventional complete dentures have a positive impact on oral health-related quality of life and satisfaction of edentulous patients.

Within the field of orthodontics there is long-standing recognition that malocclusion is definitely associated with poor OHRQoL. Although OHRQoL may be compromised during the first month of fixed orthodontic appliance therapy, it can be considerably improved at the end of whole course of treatment [4]. In order to investigate the effects of orthodontic treatment on “OHRQoL,” most researchers monitored various time points during fixed orthodontic appliance therapy such as 1 week, 1 month, 3 months, 6 months and 12 months. The advantages of this method are its simplicity for clinicians to decide when to evaluate the oral condition of patients, its convenience for research workers to record the complicated data and its sensitivity to reflect details at some point. However, it has long been accepted that comprehensive orthodontic treatment differs from most other medical interventions in that it has clear stage of clinical treatment including alignment and leveling, space closure and finishing. Therefore, greater understanding of how OHRQoL change over the three-stage process and whether or not OHRQoL of patients with different classifications of malocclusion consistent is very important in orthodontic care. In addition, although it has long been known that OHIP-14 has 7 conceptualized domains, previous studies unilaterally attached importance to aggregate score and ignored details of certain domain. Hence, exploring variations of each domain throughout the treatment process should be emphasized instead of being neglected. These information are useful to inform patient about the likely consequences of undergoing orthodontic treatment to their lives and thus can give them realistic expectations of treatment.

The aims of this study were, first, to investigate the responses of patients with Class I, Class II, Class III malocclusion to comprehensive orthodontic treatment in terms of oral health-related quality of life respectively, and second, to explore relationships between OHIP scores and clinical stage among groups with different Angle classification, and third, to characterize changes in each domain resulting from every treatment stage.

## Methods

### Sampling

The sample comprised of 90 patients who had registered for orthodontic treatment at the Department of orthodontics at Stomatology Affiliated Hospital of Shan Dong University. The inclusion criteria were non growing patients (aged15 and older) rated as having a need for comprehensive fixed orthodontic treatment by the consulting orthodontists. Exclusion criteria included patients with cognitive disorders or chronic medical conditions, those who had previously received any type of orthodontic treatment, and those with craniofacial anomalies such as cleft lip and palate, dental caries, or periodontal diseases, syndromes, facial deformities due to trauma or congenital malformation, patients who were proposed to receive other types of orthodontic appliances aside from conventional labial appliance treatment (ie, lingual orthodontic appliance or Invisalign). Patients meeting the inclusion criteria were divided into 3 treatment groups based on the type of Angle classification:

- Group1: patients with skeletal class I jaw relationship, the occlusion was an Angle Class I molar relationship, a straight facial profile, dentition crowding from moderate to severe, relieving denture crowding by extraction of 4 first premolars.
- Group2: patients with skeletal class II jaw relationship, diagnosed as Angle Class II division 1 malocclusion, excessive protrusion of maxillary incisors, at least 5 mm of overjet and 5 mm of overbite, no or slight maxillary crowding and slight or moderate mandibular crowding, a convex facial profile. Microscrew implants were used for the retraction of maxillary anterior and intrusion of the incisors. Extraction of the upper first premolars and lower first premolars were carried out for the purpose of camouflaging the anteroposterior skeletal discrepancy and obtaining a harmonious facial profile.
- Group3: patients with mild skeletal Class III relationship (−4° ≤ ANB ≤ 0°), Angle Class III molar relationship bilaterally, no or mild crowding. Mandibular and maxillary third molars were extracted before treatment, if presented. All of the participants were treated with MEAW and long Class III elastics from the upper second molar.

### Ethical considerations

Our research was conducted in full accordance with the World Medical Association Declaration of Helsinki and local legislation. The study protocol was reviewed by institutional Ethics Committee of school of dentistry, Shan Dong University and was granted ethical clearance. Informed consent were obtained from each patient to guarantee their cooperation in this study.

### Translation and adaption of the OHIP-14 inventory

The short form of the oral health impact profile (OHIP-14) consists of 14 items covering 7 domains [5, 6]: functional limitation, physical pain, psychological discomfort, physical disability, psychological disability, social disability, and handicaps. Each item is scored on a 5-point scale: 0, never; 1, hardly ever; 2, occasionally; 3, fairly often; and 4, very often or every day. Total OHIP-14 score can range from 0 to 56, and domain scores can range from 0 to 8. The baseline data (T0) of 81subjects were finished before banding and bonding of comprehensive orthodontic treatment. In subsequent research, subjects were monitored at various times during comprehensive orthodontic therapy: 1 after alignment and leveling (T1), after correction of molar relationship and space closure (T2), after finishing (T3) .

### Statistical analysis

The domain scores of OHIP-14 were obtained by summating responses to 2 corresponding items, and overall scores were derived by summating domain scores. A higher score represents poor OHRQoL. Since the data did not follow normal distribution, nonparametric tests were used in the data analysis. Friedman two way ANOVA was used to test the significant difference in OHIP-14 scores during the study period. OHIP-14 scores(overall and domain level) of adjacent stages were compared with the Wilcoxon signed rank test: T0 compared with T1, T1 compared with T2 and T2 compared with T3 to determine during what periods of treatment there were statistical difference in OHIP-14 scores. The demographic characteristics of participants and the comparison of treatment periods among three groups was analyzed by chi-square test and Friedman 2-way ANOVA respectively. The power of the samples were also recorded. The higher the power value, the more likely the test reject the null hypothesis when it is false. Power can also indicate the sample size required such that an effect of a given size is reasonably likely to be detected. Given that the statistical analysis of this research involves many analyses, a Bonferroni correction with *P* < 0.005 was used to declare significance. IBM SPSS version 16.0 software (IBM Corp, Armonk, NY, USA) was used for the processing and analysis of data.

## Results

Nine patients failed to comply with treatment and complete the questionnaires at one or more of the four observational points of the research. Thus, the overall response rate was 90 % (81/90). The missing data was distributed among former two groups (4 patients in group 1 and 5 patients in group 2). The demographic characteristics of participants are summarized in (Table 1). There were no significant differences among 3 groups in gender, age and treatment period (Table 2).

**Table 1:** Demographic characteristics of participants in three groups

Variable	Class I group	Class II group	Class III group	*p*-Value
	*N* = 35	*N* = 32	*N* = 14	
Gender				
Male	17	15	8	*P* > 0.05 (NS)
Female	18	17	6	*P* > 0.05 (NS)
Age				
15–20	20	19	9	*P* > 0.05 (NS)
20–25	15	13	5	*P* > 0.05 (NS)

*p*-values calculation was done using chi-square test; NS: not significant

**Table 2:** Comparison of time periods of 3 groups during orthodontic treatment at 3 stages (months)

Clinical stage	Class I group	Class II group	Class III group	P*
	Mean(SD)	Mean(SD)	Mean(SD)	
T0-T1	7.11(1.71)	8.13(0.25)	7(1.05)	*P* > 0.05 (NS)
T1-T2	9.05(1.2)	9.11(0.95)	8.04(1.35)	*P* > 0.05 (NS)
T2-T3	5.22(0.58)	5.43(0.77)	5.31(1.27)	*P* > 0.05 (NS)

Friedman 2-way ANOVA; P*>: level of significance;NS: not significant.

For the overall OHIP-14 score, classes I (*n* = 35), II (*n* = 32) and III (*n* = 14) showed significant decrease (*P* < 0.001) during the study period. Significant reductions (*P* < 0.001) were also observed in all seven OHIP-14 domains of three groups except for social disability in class I and class II, Handicap in class II and class III (*P* > 0.05) (Table 3).

**Table 3:** Comparison of means of overall and domain scores during orthodontic treatment at 4 time points (*n* = 81)

	T0	T1	T2	T3	P*
Domain	Mean (SD)	Mean (SD)	Mean (SD)	Mean (SD)	
All; OHIP-14					
Class I(*n* = 35)	15.32(1.24)	8.92(0.76)	5.21(0.78)	3.23(0.52)	<0.001^*^
Class II(*n* = 32)	16.42(1.03)	11.88(1.32)	6.54(0.73)	3.12(0.56)	<0.001^*^
Class III(*n* = 14)	17.11(1.13)	12.31(0.92)	5.64(0.89)	2.98(0.62)	<0.001^*^
1 Functional limitation					
Class I	1.77(0.97)	1.14(0.55)	0.66(0.53)	0.54(0.50)	<0.001^*^
Class II	1.75(0.76)	1.23(0.59)	0.63(0.55)	0.50(0.50)	<0.001^*^
Class III	1.57(0.76)	1.14(0.66)	0.50(0.51)	0.50(0.51)	<0.001^*^
2 Physical pain					
Class I	1.60(0.77)	1.23(0.59)	0.60(0.49)	0.57(0.50)	<0.001^*^
Class II	2.09(1.02)	1.19(0.69)	0.69(0.64)	0.50(0.52)	<0.001^*^
Class III	1.43(0.54)	1.07(0.61)	0.50(0.51)	0.57(0.51)	<0.001^*^
3 Psychological discomfort					
Class I	3.94(1.29)	1.63(0.49)	0.97(0.61)	0.63(0.49)	<0.001^*^
Class II	3.56(1.45)	3.44(1.39)	1.53(0.56)	0.53(0.50)	<0.001^*^
Class III	3.93(1.38)	2.71(1.06)	1.29(0.91)	0.71(0.72)	<0.001^*^
4 Physical disability					
Class I	2.57(1.17)	1.43(0.79)	0.97(0.66)	0.63(0.49)	<0.001^*^
Class II	3.56(1.13)	1.56(0.54)	1.06(0.69)	0.56(0.50)	<0.001^*^
Class III	4.14(1.46)	2.21(1.25)	1.00(0.87)	0.36(0.49)	<0.001^*^
5 Psychological disability					
Class I	4.26(1.31)	1.83(0.51)	0.91(0.45)	0.49(0.57)	<0.001^*^
Class II	3.13(1.51)	3.47(1.39)	1.59(0.56)	0.66(0.60)	<0.001^*^
Class III	4.50(1.02)	3.14(1.17)	1.29(0.76)	0.43(0.54)	<0.001^*^
6 Social disability					
Class I	0.63(0.54)	0.56(0.78)	0.69(0.83)	0.34(0.42)	0.11 (NS)
Class II	0.66(0.41)	0.42(0.67)	0.53(0.507)	0.31(0.471)	0.17 (NS)
Class III	0.93(0.47)	1.36(0.84)	0.71(0.61)	0.57(0.51)	<0.001^*^
7 Handicap					
Class I	1.14(0.69)	1.29(0.75)	0.69(0.58)	0.46(0.50)	<0.001^*^
Class II	0.61(0.21)	0.66(0.48)	0.75(0.76)	0.54(0.49)	0.36 (NS)
Class III	0.48(0.73)	0.41(0.32)	0.36(0.49)	0.32(0.36)	0.57 (NS)

Friedman 2-way ANOVA; *P* ^*^: level of significance;NS,not significant; ^*^significant at *P*<0.001

**Fig. 1:**
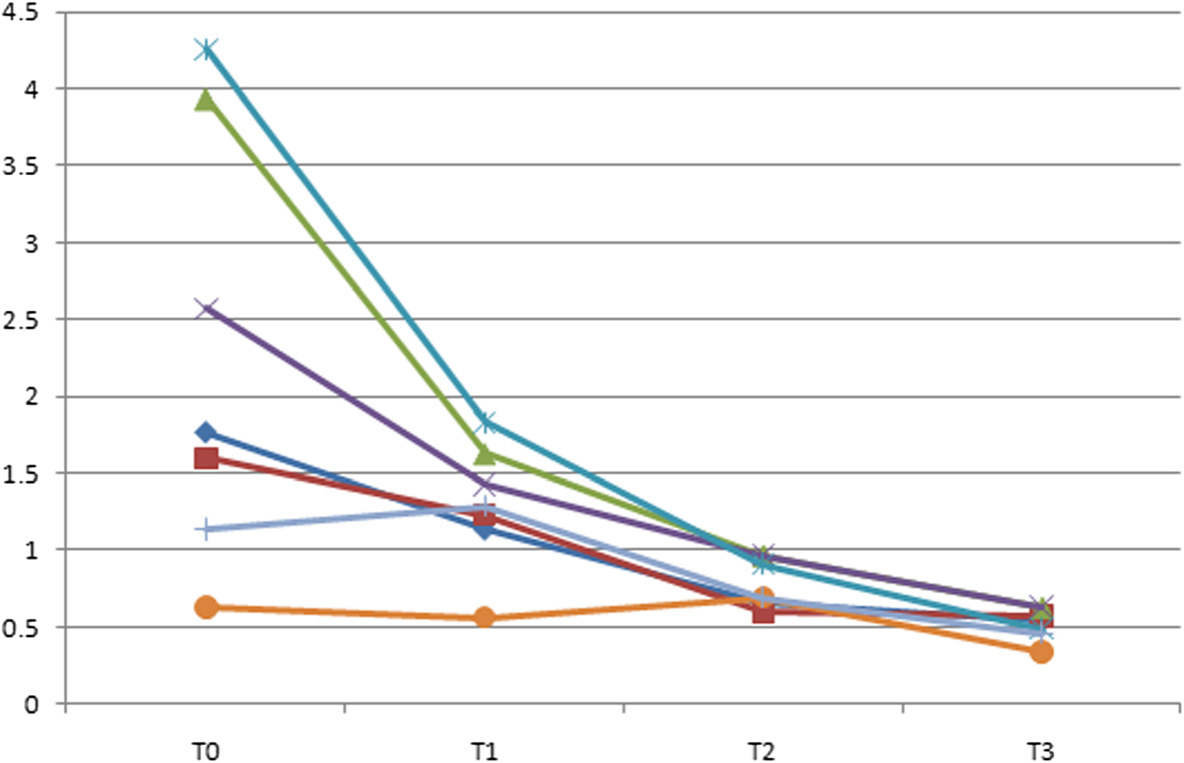
Median domain scores in Class I group at 4 different time points

**Fig. 2:**
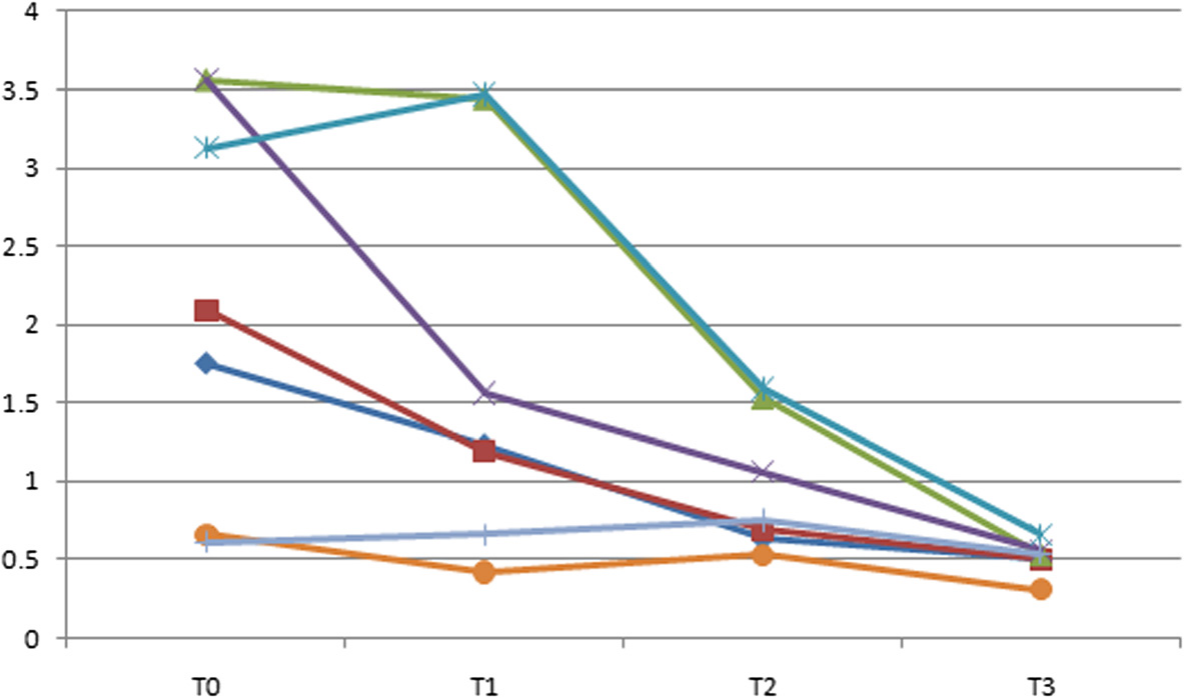
Median domain scores in Class II group at 4 different time points

**Fig. 3:**
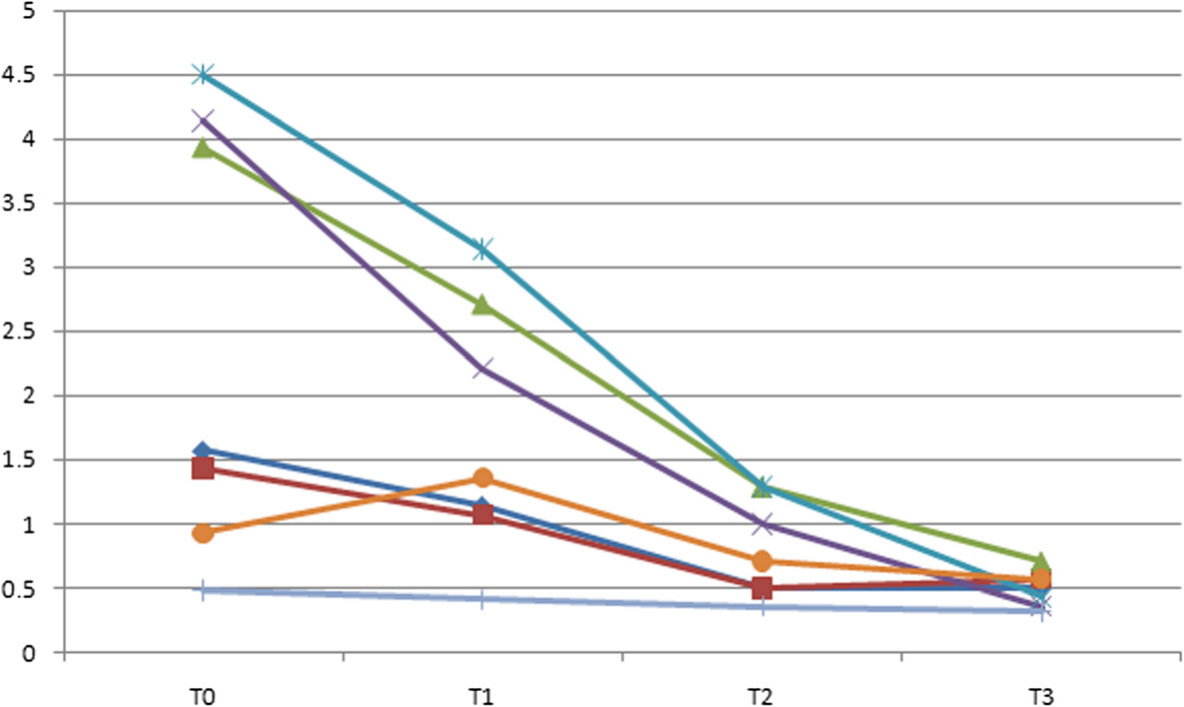
Median domain scores in Class III group at 4 different time points.  Functional Limitation;  Physical pain;  Psychological discomfort;  Psychological disability;  Physical disability;  Social disability;  handicap

In the class I group, psychological discomfort score and psychological disability scores were lower at T1 compared with T0 (*P* < 0.005), whereas there was no significant reduction between T2 and T3 (*P* > 0.005) (Table 4). Physical disability score were lower at T1 compared with T0 (*P* < 0.005), lower at T2 compared with T1 (*P* < 0.005), whereas there were no significant reduction at T3 compared with T2 (*P* > 0.005). Functional limitation and physical pain scores were significantly lower at T2 compared with T1 (*P* < 0.005), though there were no significant difference between T0 and T1 (*P* > 0.005), T3 and T2 (*P* > 0.005) (Fig. 1). In the comparisons between adjacent time points during treatment of class II malocclusion, psychological discomfort score and psychological disability score were lower at T2 compared with T1 (*P* < 0.005). Physical disability, functional limitation and physical pain scores at T1 were significantly higher than the scores at T0 (*P* < 0.005), whereas there were no significant reduction between the scores at T2 and T1, T3 and T2 (*P* > 0.05) (Fig. 2). With respect to class III group, there were significant decreases in psychological discomfort, psychological disability and social disability scores between T1 and T0 (*P* < 0.005), T2 and T1 (*P* < 0.005), T3 and T2(*P* < 0.005). At T2 compared with T1, there were significant decreases in functional limitation score, physical pain score and social disability score (Fig. 3).

**Table 4:** Comparison of differences between adjacent treatment periods(*n* = 81)

	T1-T0	Power	T2-T1	Power	T3-T2	Power
Domain	Mean (SD)		Mean (SD)		Mean (SD)	
All; OHIP-14						
Class I(*n* = 35)	−6.4(2.31)*	1.000	−3.71(2.71)*	1.000	−1.98(1.43)*	0.996
Class II(*n* = 32)	−4.54(2.42)*	1.000	−5.34(1.97)*	1.000	−3.42(1.62)*	1.000
Class III(*n* = 14)	−4.8(2.22)*	1.000	−6.67(1.77)*	1.000	−2.66(1.56)*	1.000
1 Functional limitation						
Class I	−0.38(0.37)	0.284	−0.63(0.87)*	0.583	−0.12(0.55)	0.124
Class II	−0.56(0.88)*	0.428	−0.31(0.29)	0.213	−0.13(0.47)	0.132
Class III	−0.43(0.78)	0.356	−0.64(0.56)*	0.596	0.11(0.58)	0.115
2 Physical pain						
Class I	−0.37(0.82)	0.276	−0.63(0.58)*	0.513	−0.03(0.43)	0.055
Class II	−0.92(1.01)*	0.826	−0.38(0.64)	0.311	−0.19(0.42)	0.192
Class III	−0.36(0.66)	0.272	−0.37(0.57)	0.297	0.07(0.70)	0.086
3 Psychological discomfort						
Class I	−2.31(1.46)*	1.000	−0.36(0.35)	0.264	−0.37(0.43)	0.276
Class II	−0.12(1.41)	0.137	−1.91(1.42)*	0.999	−1.01(0.53)	0.921
Class III	−1.22(1.24)*	0.982	−1.42(1.05)*	0.982	−0.58(0.81)*	0.513
4 Physical disability						
Class I	−1.14(1.27)*	0.899	−0.66(0.36)*	0.612	−0.64(0.51)	0.586
Class II	−2.01(1.16)*	1.000	−0.35(0.46)	0.253	−0.50(0.42)	0.489
Class III	−1.93(1.73)*	0.995	−1.21(0.81)*	0.953	−0.64(0.31)*	0.552
5 Psychological disability						
Class I	−2.43(1.51)*	1.000	−0.32(0.47)	0.265	−0.42(0.57)	
Class II	0.34(1.29)	0.263	−1.88(1.63)*	1.000	−0.93(0.52)*	0.856
Class III	−1.36(1.01)*	0.965	−1.85(1.07)*	1.000	−0.86(0.48)*	0.812
6 Social disability						
Class I	−0.07(0.47)	0.075	0.13(0.73)	0.129	−0.35(0.46)	0.298
Class II	−0.24(0.39)	0.152	0.11(0.64)	0.105	−0.22(0.67)	0.142
Class III	0.43(0.50)	0.332	−0.65(0.71)*	0.612	−0.14(0.58)	0.121
7 Handicap						
Class I	0.15(0.22)	0.135	−0.21(0.69)	0.117	−0.23(0.38)	0.152
Class II	0.05(0.61)	0.075	0.09(0.62)	0.101	−0.21(0.44)	0.139
Class III	−0.07(0.38)	0.092	−0.05(0.46)	0.065	−0.04(0.39)	0.067

*p values obtained from Wilcoxon signed rank test and adjusted by Bonferroni correction

*p < 0.005

## Discussion

OHRQoL is a relative concept based on subject’s own experiences and perception. Thus it is important to apply a reliable and valid instrument to assess patients’ OHRQoL in clinical practice. Both the Oral Impacts on Daily Performance (OIDP) [7] and OHIP-14 are the two most widely used indicators in evaluating Oral Health-Related Quality of Life [8]. In spite of the eight-item OIDP has proven reliable and appropriate measure to assess oral health status, there is less evidence on whether or not it is responsive to detect OHRQoL improvements and deteriorations in comprehensive orthodontic treatment. The Chinese version OHIP-14 was chosen since it was one of most commonly and sensitive measures in assessing OHRQoL changes in orthodontic treatment [9–11]. Although few investigators reported that the OHIP-14 and OIDP performed equally well, many studies have shown that OHIP-14 emerged as the superior measure with respect to construct validity and content validity due to its sensitivity towards less severe impacts [12–15]. It is for these reasons that the Chinese version OHIP-14 was chosen as research tool in our study.

In terms of changes of overall scores, research has shown that in the initial period, from one week to one month, there was a transient and significant deterioration in OHIP scores [16]. It is generally recognized that insertion of the fixed appliance places a burden to patient’s OHRQoL in the early phase of treatment. Considering that this deterioration extensively exists in initial period of comprehensive orthodontic treatment among different classifications of malocclusion, the initial period (one week to one month after the insertion of fixed appliance) hasn’t been included in our study. Most of the orthodontic literature concentrates on longitudinal analysis of the overall OHIP-14 score when evaluating the effect of orthodontic treatment on quality of life, with scant research on some inherent difference in each domain of OHIP-14. In general, improvements in appearance caused by orthodontic treatment are associated with an improvement in psychological status [17]. With respect to psychological discomfort and psychological disability, statistically significant changes were observed in patients undergoing comprehensive orthodontic treatment. However, our results indicate that these changes do not follow the same pattern among patients with different malocclusion. When analyzing the types of malocclusion in relation to the psychological discomfort and psychological disability domains evaluated by OHIP-14, this study found that patients with class I malocclusion obtained significant improvement from comprehensive orthodontic treatment only after alignment and leveling, while Class III patients benefited in all stages during treatment. Although Class II patients showed no significant benefits regarding psychological discomfort and psychological disability domains in first stage, domain scores showed an apparent decline during space closure stage. In general, there are three reasons for class II patients to seek orthodontic treatment: excessive incisor protrusion, convex facial profile and lip prominence. At the stage of space closure, microscrew implants were used to guarantee maximum retraction of upper anterior teeth. In the process of retraction, there is a continuous improvement in psychological aspect, with domain scores decreasing significantly, indicating that class II patients benefits the most from the stage of space closure. However, for class I patients who have severe or moderate dentition crowding, the goal of first phase of treatment was to bring malaligned teeth into aligned, indicating that the stage of alignment would be of value and statistically improve psychological status of class I patients.

Result from class III sample suggested that patients who had a class III malocclusion benefits in each phase of comprehensive orthodontic treatment in physical disability, psychological disability and psychological discomfort domains. Specifically, the physical aspects domain evaluates the interference of physical health problems with work and daily activity. In the present sample, improvement in physical aspects were observed throughout the entire therapeutic process of class III malocclusion, indicating that patient with class III malocclusion were better at performing routine activities than two other types of malocclusion as a consequence of comprehensive orthodontic treatment. In addition, significant improvement were also obtained for the functional limitation domain at the second stage of treatment, suggesting that by closing the space and correcting molar relationship, functional capacity: masticatory performance, speech, respiration and bite were positively affected. Isabela Branda˜o Magalha˜es [18] reported that subjects with a reduced occlusal contact area cannot pulverize their food to the same extent as subjects with more occlusal units. Fontijin-Tekamp [19] report that the number of occlusal units was the most important factor that affected the median particle size of masticatory performance. These findings might be interpreted as increased quantity of occlusal units tend to improve functional capacity of class III patients after correction of molar relationship and space closure.

Interestingly, regarding social disability domain, although progressive improvements were found from the line chart, the domain scores did not differentiate between adjacent time points during treatment in our research, in agreement with a study of health gain from orthodontic treatment [20]. In contrast, analyzing the psychosocial effects of orthognathic surgery, reported a decrease in social interaction anxiety that was related to improvement in facial esthetics [21]. Similar results have been reported in patients undergoing combined orthodontic-surgical treatment [22, 23]. One reason might be that, compared with orthodontic treatment, orthognathic surgery performed on patients can lead to an extreme change in appearance and a radical change in facial profile [24]. Therefore, changes in social disability domain were more likely to be detected in orthognathic surgery group than orthodontic group. Furthermore, it has been reported that patients with severe class III malocclusion tent to experience more social disabilities and exhibit higher levels of psychological stress in social situations than patients with mild skeletal class III malocclusion before receiving treatment [25, 26]. Hence, with regard to social disability domain, the contradiction between our findings and previous results might due to inconformity of initial status.

This study had some limitations. First, since most patients with malocclusion have strong desire and perceived need to receive orthodontic treatment, it is difficult for us to set non treatment control group. However, the shortage of non treatment control group may has impact on interpreting the results. Hence, this limitation should be acknowledged primarily. Second, The impacts of response shift and Hawthorne effect on changes in our study haven’t been excluded from results in the process of interpreting findings [27]. Third, although it would be ideal to classify patients by Angle’s classification, taking the impact of severities of malocclusion on their OHRQoL into account is preferred. The index of orthodontic treatment need (IOTN) [28], and the index of complexity, outcome, and need (ICON) [29] have been proposed to objectively quantify the severity of the various features of malocclusion. Therefore, exploring the relationship between severities of malocclusion and OHRQoL improvement obtained by comprehensive orthodontic treatment might be meaningful.

## Conclusions

1. The impact of comprehensive orthodontic treatment on patients’ OHRQoL do not follow the same pattern among patients with different malocclusion.
2. With respect to psychological discomfort and psychological disability domains, class II patients benefits the most from the stage of space closure, while class I patients benefits in the first stage (alignment and leveling) during treatment.
3. Comprehensive orthodontic treatment have little effect on patients’ social interaction anxiety, but improved occlusion and facial aesthetics do improve patients’ functional capacity and psychological well-being.

## Abbreviations

ICON: index of complexity, outcome and need
IOTN: index of orthodontic treatment need
MEAW: multiloop edgewise arch wire
OHIP: oral health impact profile
OHRQoL: oral health related quality of life
OIDP: oral impacts on daily performance
